# Individualized, Additively Manufactured Drug-Releasing External Ear Canal Implant for Prevention of Postoperative Restenosis: Development, In Vitro Testing, and Proof of Concept in an Individual Curative Trial

**DOI:** 10.3390/pharmaceutics14061242

**Published:** 2022-06-11

**Authors:** Farnaz Matin-Mann, Ziwen Gao, Jana Schwieger, Martin Ulbricht, Vanessa Domsta, Stefan Senekowitsch, Werner Weitschies, Anne Seidlitz, Katharina Doll, Meike Stiesch, Thomas Lenarz, Verena Scheper

**Affiliations:** 1 Department of Otorhinolaryngology, Head and Neck Surgery, Lower Saxony Center for Biomedical Engineering, Implant Research and Development (NIFE), Hannover Medical School, Stadtfelddamm 34, 30625 Hannover, Germany; gao.ziwen@mh-hannover.de (Z.G.); schwieger.jana@mh-hannover.de (J.S.); lenarz.thomas@mh-hannover.de (T.L.); scheper.verena@mh-hannover.de (V.S.); 2 Cluster of Excellence”Hearing4all” EXC 1077/1, 30625 Hannover, Germany; 3 Center of Drug Absorption and Transport, Department of Biopharmacy and Pharmaceutical Technology, Institute of Pharmacy, University of Greifswald, 17489 Greifswald, Germany; martin.ulbricht@uni-greifswald.de (M.U.); vanessa.domsta@uni-greifswald.de (V.D.); stefan.senekowitsch@uni-greifswald.de (S.S.); werner.weitschies@uni-greifswald.de (W.W.); anne.seidlitz@hhu.de (A.S.); 4 Institute of Pharmaceutics and Biopharmaceutics, University of Duesseldorf, 40225 Dusseldorf, Germany; 5 Clinic for Dental Prosthetics and Biomedical Materials Science, Hanover Medical School, 30625 Hannover, Germany; doll.katharina@mh-hannover.de (K.D.); stiesch.meike@mh-hannover.de (M.S.)

**Keywords:** ear canal stenosis, personalized implant, drug-eluting implant, additive manufacturing, preoperative workflow, external auditory canal

## Abstract

Postoperative restenosis in patients with external ear canal (EEC) atresia or stenosis is a common complication following canaloplasty. Our aim in this study was to explore the feasibility of using a three dimensionally (3D)-printed, patient-individualized, drug ((dexamethasone (DEX)), and ciprofloxacin (cipro))-releasing external ear canal implant (EECI) as a postoperative stent after canaloplasty. We designed and pre-clinically tested this novel implant for drug release (by high-performance liquid chromatography), biocompatibility (by the MTT (3-(4,5-dimethylthiazol-2-yl)-2,5-diphenyltetrazolium bromide) assay), bio-efficacy (by the TNF-α (tumor necrosis factor-alpha)-reduction test (DEX) and inhibition zone test (for cipro)), and microbial contamination (formation of turbidity or sediments in culture medium). The EECI was implanted for the first time to one patient with a history of congenital EEC atresia and state after three canaloplasties due to EEC restenosis. The preclinical tests revealed no cytotoxic effect of the used materials; an antibacterial effect was verified against the bacteria *Staphylococcus aureus* and *Pseudomonas aeruginosa*, and the tested UV-irradiated EECI showed no microbiological contamination. Based on the test results, the combination of silicone with 1% DEX and 0.3% cipro was chosen to treat the patient. The EECI was implantable into the EEC; the postoperative follow-up visits revealed no otogenic symptoms or infections and the EECI was explanted three months postoperatively. Even at 12 months postoperatively, the EEC showed good epithelialization and patency. Here, we report the first ever clinical application of an individualized, drug-releasing, mechanically flexible implant and suggest that our novel EECI represents a safe and effective method for postoperatively stenting the reconstructed EEC.

## 1. Introduction

Stenosis or atresia of the external ear canal (EEC) can be a congenital malformation characterized by an underdeveloped EEC, but it can also be acquired or iatrogenic. The congenital condition occurs in 1 of 10,000–20,000 live births [1,2], is bilateral in approximately one-third of patients, and could be associated with microtia or inner ear abnormalities. Acquired EEC stenosis may emerge due to exostosis, chronic EEC infection, or postoperatively [1]. Postoperative EEC stenosis following canaloplasty can be caused by bony overgrowth and soft tissue hypertrophy, i.e., scar formation [3]. Soft tissue hypertrophy is characterized by persistent inflammation and by extreme deposition of fibroblast-derived extracellular matrix proteins, especially collagen [3,4,5,6]. The current literature reveals a restenosis rate following canaloplasty as high as 50% [7]. Therefore, reducing the rate of postoperative EEC stenosis and the subsequent need for revision surgery is essential in canaloplasty. No standard guidelines are available for postoperative management after canaloplasty [3] and the management remains very challenging since none of the present therapies reliably lead to clinical success. According to international clinical recommendations on scar management, the treatment of hypertrophic scar is most successful when the scar tissue is immature but the overlying epithelium is intact [8]. Therefore, it is more important to prevent the postoperative development of a hypertrophic scar than to treat the scar tissue afterwards. Hypertrophic scars are caused by increased and persistent inflammation after tissue damage.

To date, different materials are inserted postoperatively into the EEC to apply mechanical pressure to prevent the risk of restenosis due to bony overgrowth and soft tissue hypertrophic scars [3]. The common materials used are soaked absorbable gelatin sponges (Gelfoam^®^), liquid antibiotic gauze (soaked with Bismuth Iodoform Paraffin Paste or Xeroform^TM^) and expandable ear wicks [7]. These packing materials are generally fixed in situ for one to two weeks. Other materials used and described in the literature are short-term silicone or acrylic for long-term ear molds [3,9]. Different packing materials such as gauze packing or Merocel^®^ wicks have shown to lead to increased exudation, thereby triggering inflammation and granulation, which are both known causes of canaloplasty failure [1]. Therefore, one limitation of post-canaloplasty molds are that the EEC should not be completely occluded. This does not only lead to poor healing and EEC bacterial or fungal infections, but also hampers hearing [1].

Stenting the EEC after surgery showed to be a good alternative. Hollow prostheses allow ventilation and drainage from the EEC and in the case of postoperative infections the administration of ear drops [7]. There are reports about stenting the EEC with rededicated devices such as a soft ventilation tube [10], nasopharyngeal tube [11], composite Foley catheter [12], and rubber tube [13]. Acrylic and silicone stents individualized by molding the EEC were used by Savion et al. [9] and Moon et al. [3] and were reported to offer good compliance to the stent size and width [7]. However, postoperatively not only the pressure therapy is important. Additionally, preventing the newly reconstructed EEC from infections and hypertrophic scar formation has to be considered. To date, none of the structural stents were loaded with or could release drugs for the prevention of infections.

For the prevention of EEC infections, there are currently only two US Food and Drug Administration (FDA)-approved ototopical antibiotic preparations, both of which are fluoroquinolone derivatives: 0.3% ofloxacin and 0.3% ciprofloxacin (cipro)/0.1% dexamethasone (DEX) otic suspension [14]. Cipro represents the drug of choice for treating otitis externa in children and adolescents as it has been the subject of extensive investigation [15]. DEX is a synthetic corticosteroid that has anti-inflammatory and immunosuppressive activity [16]. The application of DEX has been widely reported to reduce a number of mediators that promote fibrosis and reduce the fibrotic sheath formation [17,18]. DEX exerts pleiotropic activity by inhibiting the bacterial lipopolysaccharide (LPS)-induced cytokine production of tumor necrosis factor-alpha (TNF-α), thereby modulating innate immunity in activated macrophages [19]. For this reason, in the current study, we applied DEX and cipro via extended release from the EEC implant (EECI) into the EEC.

Taking the hearing aids as an example, it is known how highly individualized the shape of the EEC is and how important it is to consider this in order to achieve an adequate fit in the canal, and thus prevent restenosis. The stent material should be mechanically flexible so it can be surgically inserted with ease to avoid additional trauma of the ear canal due to implantation, but should also be resistant to mechanical pressure of the surrounding tissue. Concurrent pharmacotherapy to avoid hypertrophic scar formation and infection with the abovementioned substances could be a useful addition to simple stenting. A promising approach to addressing these requirements, which has not yet been applied to the EEC region yet, is the use of additively manufactured drug-loaded individualized EECI.

We demonstrated the preparatory work performed to ensure the safety and efficacy of this novel implant. We designed and three dimensionally (3D) printed an EECI made from silicone containing DEX and cipro. The in vitro test results as well as an individual curative trial, where the EEC stenting is accomplished through the use of this patient-individualized, drug-releasing EECI are reported.

To our knowledge, our study is the first to manufacture, pre-clinically test, and clinically implant a 3D-printed, mechanically flexible, drug-releasing implant that was individualized with regard to the patient’s anatomy.

## 2. Materials and Methods

### 2.1. Case Presentation

**Figure 1:**
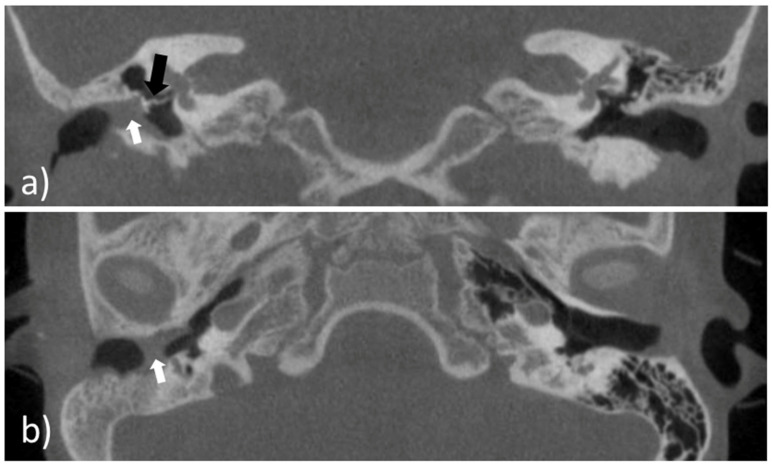
Cone beam computed tomography imaging of the temporal bone of the patient. (**a**) Coronal view of the external ear canal with the white arrowhead pointing to the restenosis; an aerated middle ear space and a middle ear partial ossicular replacement prosthesis (black arrowhead); (**b**) axial view of the external ear canal with the partial restenosis (white arrowhead).

An eight-year-old girl with the diagnosis of unilateral congenital microtia and atresia of the EEC on the right side presented to our tertiary care hospital in January 2021. She had a patient history of multiple surgeries including reconstruction of the bony EEC and tympanoplasty type IIIa on the right in May 2018 and two revision canaloplasties with tympanic membrane (TM) reconstruction after restenosis of the EEC on the right side in January 2019 and September 2019. The clinical exam showed a partial restenosis of the surgically constructed EEC on the right side. A cone beam computed tomography imaging of the temporal bone (CBCT; xCAT, Xoran Technologies, Inc., Ann Arbor, MI, USA) (Figure 1) demonstrated a partial restenosis of the EEC on the right, but an aerated middle ear space, a middle ear partial ossicular replacement prosthesis (PORP), and an aberrant facial nerve course.

We decided to perform ear canal revision surgery and implantation of an individualized drug-releasing EECI to prevent postoperative restenosis.

### 2.2. Preclinical Testing of the External Ear Canal Implant 

The printed and drug containing implant material was tested for drug release (by high-performance liquid chromatography (HPLC)), biocompatibility (by MTT assay (3-(4,5-dimethylthiazol-2-yl)-2,5-diphenyltetrazolium bromide assay), bio-efficacy (by TNFα-reduction test (DEX), inhibition zone test (for cipro)), and microbial contamination. For this purpose, an EECI model and different test specimens were printed.

### 2.3. Manufacturing the External Ear Canal Implant and Test Specimen 

#### 2.3.1. Segmentation of the External Ear Canal and Design of the Implant Model 

**Figure 2:**
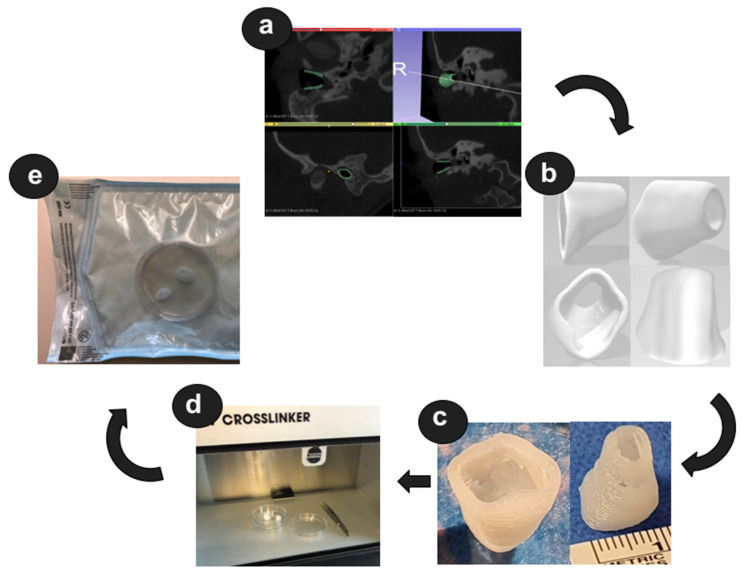
Development of a 3D-printed external ear canal implant (EECI). (**a**) Manual segmentation of the area to be implanted (green); (**b**) digital model of the EECI exported as STL data; (**c**) 3D-printed EECI; (**d**) irradiation of the implant using the UV crosslinker; (**e**) irradiated implant packed in the autoclaved pouch.

Prior to the preclinical testing, the patient presented to our outpatient clinic. CBCT imaging of the temporal bone was performed (Figure 1) to capture the anatomy of the region of interest (ROI). The acquired data were saved as image slices in the DICOM (digital imaging and communications in medicine) format and the ROI was segmented manually by a trained ENT surgeon using 3D Slicer^TM^ version 4.11 (Surgical Planning Laboratory, Brigham and Women’s Hospital, Harvard Medical School, Boston, MA, USA) (http://www.slicer.org; accessed on 1 November 2021) to build a 3D reconstruction of the bony EEC. The segmentation resulted in the isolation of the ROI through a semi-automatic process based on region thresholding of the bony edges of the EEC (Figure 2a). After segmentation, the implant surface was processed by applying the surface smoothing effect with the dimensionless parameter of 0.5 in 3D Slicer^TM^. All reconstructions were transformed into a hollow object with a wall thickness of 1.5 mm and then the ends facing to the TM and the opening of the EEC were cut. Subsequently, a stereolithography (STL) file of the digital model was generated (Figure 2b).

#### 2.3.2. 3D Printing of the External Ear Canal Implant 

The STL file was loaded into the Perfactory RP software (EnvisionTEC GmbH, Gladbeck, Germany) and was sliced into 320 μm slices (80% of the needle diameter, detailed below). The resulting file was imported to Visual Machines software version 2.8.130r7 (EnvisionTEC GmbH, Gladbeck, Germany), where the model was assigned an infill with a fiber spacing of 0.4 mm and a 90° layer-to-layer rotation, and a single contour outline. The patient-specific EECI for the planned implantation as well as the EECI model for preclinical testing were 3D printed using a 3D-Bioplotter^®^ Manufacturers Series (EnvisionTEC GmbH, Gladbeck, Germany), equipped with a low-temperature printing head operated at a pneumatic pressure of 5 bar and a UV Curing Head at 365 nm. Medical-grade UV silicone (60A MG, BIO-83-6001, EnvisionTEC, silicone elastomer curing at 365 nm, USP Class VI)) with its silicone catalyst (catalyst compound, EnvisionTEC) was prepared in a ratio of 50:1 using the Speedmixer™ DAC 150.1 FVZ (Hausschild & Co.KG, Hamm, Germany) for two minutes operated at 3500 rpm. To this silicone elastomer, 1% DEX (Caesar & Lorentz GmbH, Hilden, Germany) and 0.3% cipro (Sigma Aldrich, Hamburg, Germany), both in powder form, were added using the Speedmixer™ again for two minutes operated at 3500 rpm. The silicone/drug mix was loaded into the low-temperature head attached with a 400 μm dispensing needle tip (Nordson Australia Pty Ltd., Sydney, Australia) and printed at 27 °C at a movement speed of 2 mm/s. The silicone was crosslinked layer-by-layer using the UV light source. Finally, the EECI was irradiated using UV-light (1800 × 100 µJ/cm²) from above and below—the opening of the EECI towards the TM and the EEC as shown in Figure 2c—for 30 min in a UV-Crosslinker equipped with five 8-watt 254 nm bulbs (SpectroLinker XL-1000; Spectroline, Westbury, NY, USA) to eliminate microbiological contamination (Figure 2d) and subsequently transferred to an autoclaved pouch (Self Seal Sterilization Pouch, 134 × 280 mm, Henry Schein Inc., Melville, NY, USA) (Figure 2e) using sterile gloves and forceps.

Beside the EECI model, different test specimens and formulations were manufactured. Three different formulations were prepared using the Speedmixer™ DAC 150.1 FVZ (Hausschild & Co.KG, Hamm, Germany) and printed for the preclinical testing (biocompatibility (MTT assay), bio-efficacy (TNFα-reduction test (DEX), and inhibition zone test (cipro)): test specimens with the dimensions of 0.5 × 0.5 × 0.1 cm as well as discs with a diameter of 0.5 cm and a height of 0.5 cm of pure UV–silicone, UV–silicone containing 1% DEX and 0.3% cipro, and UV–silicone containing 2% DEX and 0.3% cipro. The test specimens were printed in the same way as the EECI described above.

### 2.4. Eluate Preparation for Biocompatibility and Bio-Efficacy Tests

To test the biocompatibility and bio-efficacy of the 3D-printed drug-containing UV–silicone, eluates were generated by incubating the samples (n = 3 per condition) in saline (600 µL NaCl 0.9%, B. Braun, Melsungen, Germany) in a 48-well plate (Nunc, Thermo Fisher Scientific, Waltham, MA, USA) for 48 h in an incubator (CB150; Binder, Tübingen, Germany; 37 °C, 5% CO_2_, 95% humidity).

### 2.5. Biocompatibility Testing

A potential cytotoxic effect of the manufactured implant was analyzed by MTT assay (3-(4,5-dimethylthiazol-2-yl)-2,5-diphenyltetrazoliumbromid assay, substances from PanReac AppliChem GmbH, Darmstadt, Germany) using NIH/3T3 mouse fibroblasts (German Collection of Microorganisms and Cell Cultures GmbH, Braunschweig, Germany) (passage 3 to 10) (ATCC-Number: CRL-1658). Cells were seeded in a density of 1.5 × 10^4^ cells/mL in a 96-well plate (Thermo Fisher Scientific, Roskilde, Denmark) and cultured in medium consisting of DMEM (Dulbecco’s Modified Eagle’s Medium), 10% FCS (fetal calf serum; both from Bio & Sell GmbH, Feucht/Nürnberg, Germany), and 1% penicillin/streptomycin (Biochrom GmbH, Berlin, Germany) in a humidified atmosphere of 5% CO_2_/95% air at 37 °C. After 24 h, the culture medium was discarded and replaced by 100 µL fresh medium. An additional 50 µL saline was added to the negative control (NC) and the positive control (PC) while 50 µL eluate of each silicone sample was added to the residual wells. The PC was additionally treated with DMSO (0.1%, dimethyl sulfoxide, AppliChem GmbH, Darmstadt, Germany) to induce toxic effects on the cells. Each condition was performed in three-fold repetition, resulting in n = 3 for NC and PC and a total of n = 9 for the three samples per silicone group. Cells were cultured for additional 24 h. Then, the medium was discarded and replaced by solutions for the MTT assay and, finally, the optical density (O.D.) was read at a wavelength of 570 nm using a microplate reader (Gen5 2.06.Ink, BioTek Synergy™ H1HyBrid Reader, Santa Clara, CA, USA). Measurement of blank wells without cells was included for blank correction of the O.D. The relative cell viability was calculated in percent by dividing the blank-subtracted O.D. of the test groups by the blank-subtracted O.D. of the NC and multiplying the result by 100. Based on the ISO guideline 10993-5:2009 [20] for biological evaluation of medical devices, a cell viability below 70% was judged as cytotoxic. An unpaired *t*-test was performed with GraphPad Prism^®^ version 8.4.3 (GraphPad Prism Software Inc., La Jolla, CA, USA) to detect differences between the groups.

### 2.6. Bio-Efficacy of DEX: TNFα-Reduction Test

The bio-efficacy of the drug-containing 3D-printed UV–silicone was tested in a TNFα-reduction assay. The principle of this assay is to stress the cells in order to stimulate the production of TNFα by LPS treatment. The incorporated and released DEX should lead to a reduction in the production of TNFα. Cells of the DC2.4 mouse dendritic cell line (DCs) (Sigma-Aldrich, St. Louis, MO, USA, LOT:3093896) were seeded at a density of 0.5 × 10^4^ cells/well in a 96-well plate (Thermo Fisher Scientific, DK-4000, Roskilde, Denmark) and cultured for 48 h (see above; incubator, humidified atmosphere of 5% CO_2_/95% air at 37 °C) until a density of 70–80% was achieved. The culture medium consisted of RPMI 1640 medium (Sigma-Aldrich, St. Louis, MO, USA) supplemented with non-essential amino acids (1 mmol/L, Sigma-Aldrich, St. Louis, MO, USA) and 10% FCS (Bio & Sell GmbH, Feucht, Germany). The cells were supplied with 100 µL fresh medium including 0.5 µg LPS/mL (Sigma Aldrich, St. Louis, MO, USA) to induce stress, except for the NC lacking LPS to detect the baseline TNFα production of the DC2.4 cells. The PC was treated with LPS only to determine the TNFα level of stressed cells and a DEX-control (DEX; 60 µM in 100 µL; i.e., 23.5 µg/mL) was included as reference for the anti-inflammatory effect as a known concentration of DEX. Then, 50 µL of each eluate was added to the cells and the controls (NC, PC, DEX-control) were treated with 50 µL saline by the same procedure. The controls and each of the three eluates were tested in triplicate in the DC culture. After cultivation for an additional 24 h, the supernatant was collected and pooled for the three wells of each condition to be able to perform dilution series in the subsequent analysis for TNFα-concentration by using an enzyme-linked immunosorbent assay (ELISA) kit (Mouse TNF-α ELISA Kit PicoKine, Boster Biological Technology, Pleasanton, CA, USA). Following replicates were analyzed per condition: n = 3 for NC, PC, and pure silicone and n = 9 for silicone DEX 1% and cipro 0.3%, silicone DEX 2%, cipro 0.3%. ELISA detection was performed in accordance with the manufacturer’s instructions. The cells were fixed with 4% formalin (Merck Millipore, Burlington, MA, USA) and the nuclei were labeled with DAPI (4′,6-diamidin-2-phenylindol; 1:100; Merck Millipore, Burlington, MA, USA) for 10 min at room temperature. The O.D. for the cell density was subsequently determined with the plate reader (excitation/emission of 358/461 nm) to normalize the measured TNFα-concentration to the cell number of the different conditions. Data were analyzed with GraphPad Prism^®^ version 8.4.3 (GraphPad Prism Software Inc., La Jolla, CA, USA) for significant differences using the Kruskal–Wallis statistic with Dunn’s multiple comparison test, to take the small sample size into account.

### 2.7. Bio-Efficacy Test of Cipro: Microbiological Inhibition

The bacteria-free areola test was conducted on the basis of the DIN 58940-3 norm in N = 3 × 3 replicates. *Staphylococcus aureus* (DSM 799, German Collection of Microorganisms and Cell Cultures GmbH, Braunschweig, Germany) and *Pseudomonas aeruginosa* (ATCC BAA-47, American Type Culture Collection, Manassas, VA, USA) were routinely stored as glycerol stocks at −80 °C and pre-cultivated in tryptone soy broth (Oxoid Limited, Hampshire, UK) supplemented with 10% yeast extract (Carl Roth GmbH & Co. KG, Karlsruhe, Germany) for 18 h agitating under aerobic conditions at 37°C. For bacteria-free areola test, bacteria were harvested by centrifugation and adjusted to a final optical density at 600 nm of 0.01 in phosphate-buffered saline (Biochrome GmbH, Berlin, Germany). Each bacterial suspension was streaked on half a tryptone soy broth +10% yeast extract agar plate using a sterile swab. In the middle of each plate, in contact to both bacterial strains, a sample disc was placed. The plates were incubated for 24 h under aerobic conditions at 37 °C. Afterwards, the radius of the bacteria-free areola was manually measured for both strains and the diameter was calculated. Data evaluation for microbiological inhibition was done using the GraphPad Prism software (version 8.4, GraphPad Prism Software Inc., La Jolla, CA, USA). Gaussian distribution was tested with D’Agostino–Pearson omnibus normality test and statistical differences with ordinary one-way ANOVA and Tukey’s multiple comparison test. Significance level was set to α = 0.05.

### 2.8. Drug Release Analysis

DEX (Euro OTC Pharma GmbH, Cologne, Germany) and cipro (Sigma-Aldrich, St. Louis, MO, USA) were used as reference for the quantification of the active ingredients for the release test. For the preparation of the phosphate-buffered saline (PBS) pH 7.4 (European Pharmacopoeia), the buffer salts disodium hydrogen phosphate dodecahydrate (AppliChem GmbH, Darmstadt, Germany), potassium dihydrogen phosphate (LaboChem international, Heidelberg, Germany), and sodium chloride (Ceasar & Loretz GmbH, Hilden, Germany) were used. For the phosphate buffer with triethylamine pH 3.0, potassium dihydrogen phosphate (LaboChem International, Heidelberg, Germany) and triethylamine (VWR Chemicals, Fontenay-sous-Bois cedex, France) were used. Ultrapure water was obtained from a Milli-Q unit (Millipore, Billerica, MA, USA).

For the drug release study, the EECI models were incubated in 2 mL PBS pH 7.4 for 24 d. The investigations were performed in 15 mL reagent tubes (Sarstedt AG & Co. KG, Nümbrecht, Germany) under shaking at 100 rpm and 37°C (IKA^®^ KS 3000 i control, IKA^®^-Werke GmbH & Co. KG, Staufen, Germany). The masses of the EECI models were determined prior to release testing in order to estimate the theoretical drug load of the implants (1% DEX and 0.3% cipro). The EECI models were placed in the pre-heated release medium and transferred to a new tube containing fresh medium after each sampling time point. The transfer of the EECI models was performed after 1 h, 6 h, 1 d, 3 d, 7 d, 10 d, 14 d, 18 d, 21 d, and 24 d. During this process, the implants were carefully blotted on cellulose between repositioning to avoid carryover. Care was taken to ensure a gentle transfer with little mechanical stress on the implants in order to avoid potential influence on the release behavior. To account for evaporative losses, the masses of the reagent tubes were determined (empty and with release medium). Thereby, the exact volume used during release was calculated. For this purpose, the density of the release medium was determined beforehand (Handheld Density Meter, Densito, Mettler Toledo, Columbus, OH, USA). Sink conditions were maintained during the experiments. The release medium samples were frozen and stored at −20 °C until analysis of drug content.

To determine the released amount of DEX and cipro, a HPLC apparatus (Shimadzu Corporation, Kyoto, Japan) consisting of a LC40B xr solvent delivery module, a SPD-M40 photodiode array detector, a DGU-403 degassing unit, a Sil-40C xr auto sampler and a CTO-40S column oven was used. A Phenomenex Kinetex C8 column (150 × 2.1 mm, 2.6 µm, equipped with an according precolumn, Phenomenex Inc., Torrance, CA, USA) was utilized for separation of the compounds. The oven temperature was set to 45 °C. The autosampler temperature was kept at 20 °C. A phosphate buffer containing triethylamine (pH 3.0, with potassium dihydrogen phosphate 17.99 mM and triethylamine 7.12 mM, eluent A) and methanol (eluent B) were used as the mobile phase. The flow rate was set to 0.4 mL/min. A binary gradient was used for the method. The detailed conditions of the used gradient of the HPLC method are listed in Table 1.

One microliter of the sample was injected (undiluted) and analyzed. The retention times were 11.4 min for DEX and 2.5 min for cipro, respectively. For quantification of the compounds, the peak areas of the obtained chromatograms at a wavelength of 254 nm for DEX and of 278 nm for cipro, respectively, were integrated. Calibration standards were prepared by diluting stock solutions of DEX and cipro in a mixture of phosphate buffer with triethylamine and methanol (ratio of 80:20 *v*/*v*), which were also injected for analysis. A linear least-squares regression was applied for the calculation of the concentration of the samples. The quantification range was between 0.41–40.81 µg/mL for DEX and between 0.12–12.24 µg/mL for cipro.

Accuracy and precision were investigated at 3 concentration levels (28.56 µg/mL, 12.24 µg/mL, 3.06 µg/mL for DEX, and 8.57 µg/mL, 3.67 µg/mL, 0.9 µg/mL for cipro) by running quality control samples that had been prepared in triplicate (coefficient of variation maximum 5%).

### 2.9. Pre-Test on Microbial Contamination

The 3D-printed EECI models were tested for the growth of microorganisms by incubation in culture media to gain an initial impression of the microbiological status of the implants. DEX (1%) loaded EECI models were printed for this test without cipro to avoid a falsification of the test results owing to the antimicrobial properties of this drug. Test samples were irradiated using UV light as described above after the printing process to reduce the initial microbial load in the same way as the EECI for application in the patient.

Under the aseptic conditions of a laminar airflow workbench, the EECI models were transferred into sterile test tubes and 5 mL of sterile culture medium was added to the UV-treated EECI models in triplicate, respectively. Two different types of culture medium, soya-bean casein digest medium (CASO Broth, Carl Roth GmbH + Co. KG, Karlsruhe, Germany) and fluid thioglycollate medium (Fluid Thioglycollate Medium, VWR International bvba/sprl, Leuven, Belgium), were used to allow the growth of a wide spectrum of microorganisms. Related to the sterility test of the European Pharmacopoeia with the direct inoculation method, the samples in soya-bean casein digest medium were incubated at 20–25 °C for 14 days and were meanwhile regularly monitored visually for turbidity or sediments as an indicator for the growth of aerobic bacteria, fungi, or yeasts. The samples in the fluid thioglycollate medium were incubated at 30–35 °C for 14 days and similarly monitored to recognize the growth of anaerobe or aerobe bacteria. Furthermore, positive and negative controls were also processed in duplicate to ensure the functionality of the test. Negative controls with pure medium were incubated under the same conditions as the EECI models to control the aseptic filling process in culture medium. As positive control, 5 mL culture medium without an EECI model was spiked with a small amount of *Bacillus subtilis* (recultivated from ATCC 6051, Leibniz Institute, DSMZ-German Collection of Microorganisms and Cell Cultures GmbH, Braunschweig, Germany) and incubated under identical conditions to verify the suitability of the culture media. Additionally, small amounts of *Bacillus subtilis* were spiked into culture medium containing an EECI model to exclude antimicrobial effects of the sample material that might have falsified the test results.

## 3. Results

### 3.1. Biocompatibility and Bio-Efficacy 

**Figure 3:**
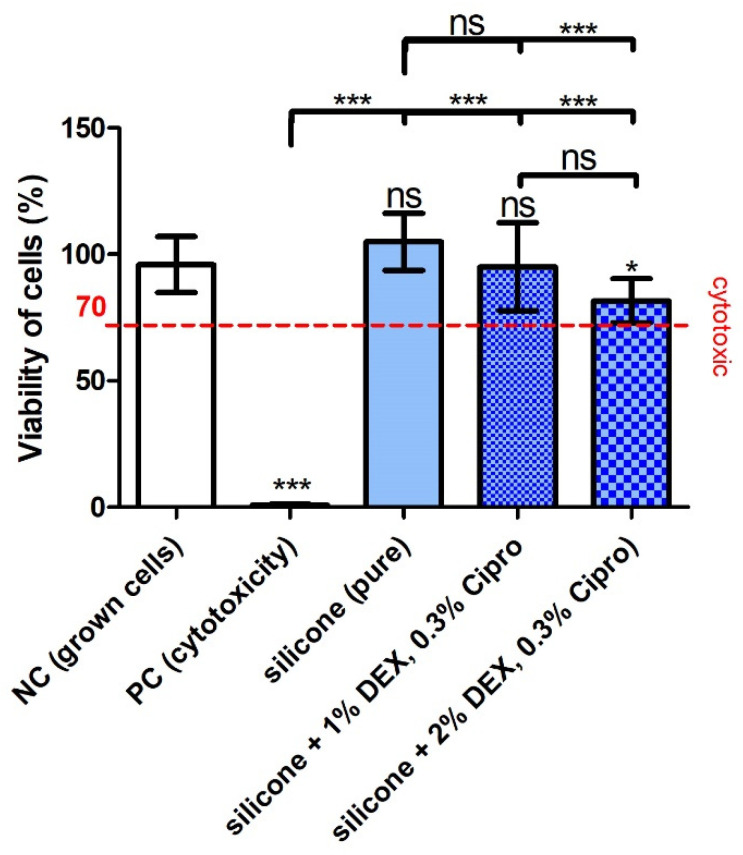
Cell viability results of fibroblasts treated with the supernatant of the different conditions. The positive control (PC) showed a massive and significant reduction of cell viability compared to the NC and silicone groups, indicating the cytotoxic effect of DMSO and proving that the test was appropriately performed. There were no significant differences detectable between the negative control (NC) and the silicone groups except for silicone with 2% dexamethasone (DEX). This higher amount of included DEX also reduced the cell viability compared to pure silicone, while this was not the case for 1% DEX. A viability of 70% (red line) is labeled to mark the cut-off for the cytotoxic effect of materials (ISO 10993-5:2009). Only the PC showed a cell viability below this mark. Data are given as mean ± standard deviation (SD) and significances are labeled with *** *p* < 0.001, * *p* < 0.05, and ns: not significant. Significances marked above the error bar refer to the NC. N = 1; n = 3 (NC, PC), n = 9 (silicone, silicone DEX 1%, cipro 0.3%, silicone DEX 2%, cipro 0.3%).

The biocompatibility of the printed samples was tested in the MTT assay (Figure 3). Compared to the NC and the silicone groups, the cell viability of the PC including the cytotoxic agent DMSO was significantly reduced, indicating the successful experimental setup. Neither the addition of eluates of the pure silicone nor of the silicone with 1% DEX and 0.3% cipro had a significant effect on the cell viability compared to the NC. In contrast, the inclusion of 2% DEX and 0.3% cipro in the silicone reduced the cell viability in comparison to the NC and the pure silicone. Regarding the cytotoxicity, the cell viability of PC (mean < 1%) was far below the 70% mark for cytotoxicity whereas the cell viability of silicone samples with and without drugs was 105 ± 4% (silicone), 95 ± 6% (silicone DEX 1%, cipro 0.3%), and 82 ± 3% (silicone DEX 2%, cipro 0.3%) and therefore clearly above the 70% mark.

**Figure 4:**
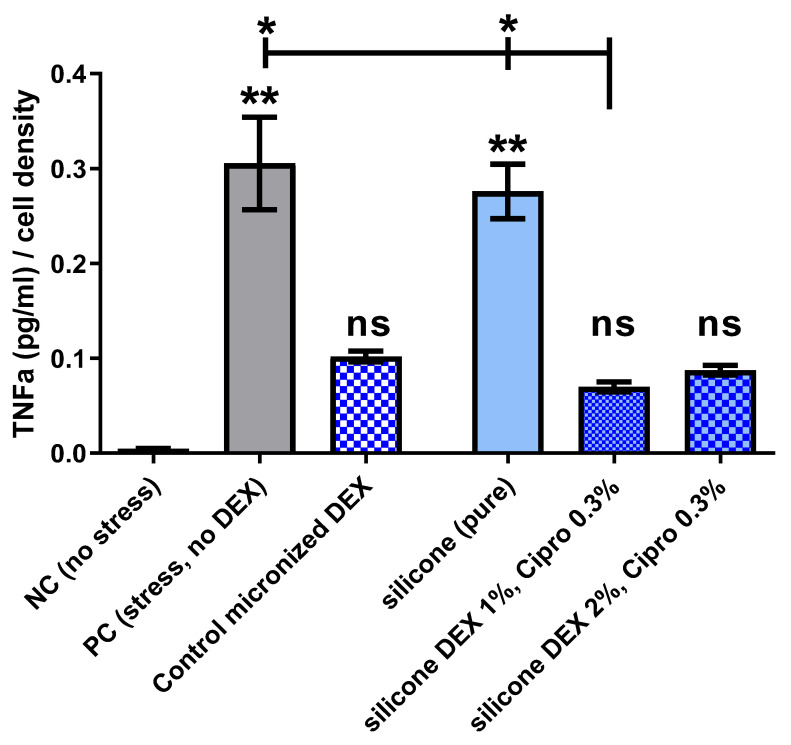
The ELISA-detected TNFα (tumor necrosis factor-alpha)-concentration normalized to the optical cell density in the dendritic cell culture of the different experimental groups is shown. Differences to the NC with basic TNFα-level of lipopolysaccharide (LPS)-unstressed cells are indicated above the bars. In the PC and for the pure silicone, the TNFα concentration was significantly increased compared to the NC, while the inclusion of DEX reduced the levels to an amount not differing significantly from the NC. There was no difference detectable between the anti-inflammatory efficacy of DEX solution added to the medium or DEX released from the silicone into the eluates of the samples. The inclusion of 1% DEX (and 0.3% cipro) in the silicone reduced the TNFα-production induced by LPS stress significantly compared to the PC and pure silicone. This significant difference was not detected for 2% DEX-releasing samples or the DEX-control even though there was no difference in TNFα reduction compared to 1% DEX-loaded samples. Data are given as the mean ± SD and significances are labeled with ** *p* < 0.001, * *p* < 0.05, ns: not significant. N = 1; n = 3 (NC, PC, silicone), n = 9 (silicone DEX 1%, cipro 0.3%, silicone DEX 2%, cipro 0.3%).

To investigate the anti-inflammatory effect of released DEX, a TNFα-reduction test (Figure 4) was conducted. Cells without stress (NC) showed a very low basic level of TNFα-production (0.005 ± 0.0007 pg/mL TNFα/cell density) while this level increased significantly with addition of LPS (PC) to amounts of 0.3 ± 0.05 pg/mL TNFα/cell density. When no drugs were included into the silicone, the amount of TNFα (0.27 ± 0.03 pg/mL TNFα/cell density) was similar to the PC and significantly increased compared to the NC. Addition of DEX to the cell culture, whether in thw form of pure DEX (DEX-control) or released from the silicone samples, reduced the TNFα amount and thus the cell stress to a level not significantly differing from the NC. This anti-inflammatory effect was highest for silicone including 1% DEX and 0.3% cipro (0.07 ± 0.005 pg/mL TNFα/cell density), which showed as only DEX-including condition a significant reduction of TNFα in the cell supernatant compared to PC and silicone without drugs. Due to the need for pooling the supernatant of the replicate wells for each condition to be able to perform dilution series for ELISA testing, some statistical scattering for these results must be taken into account.

**Figure 5:**
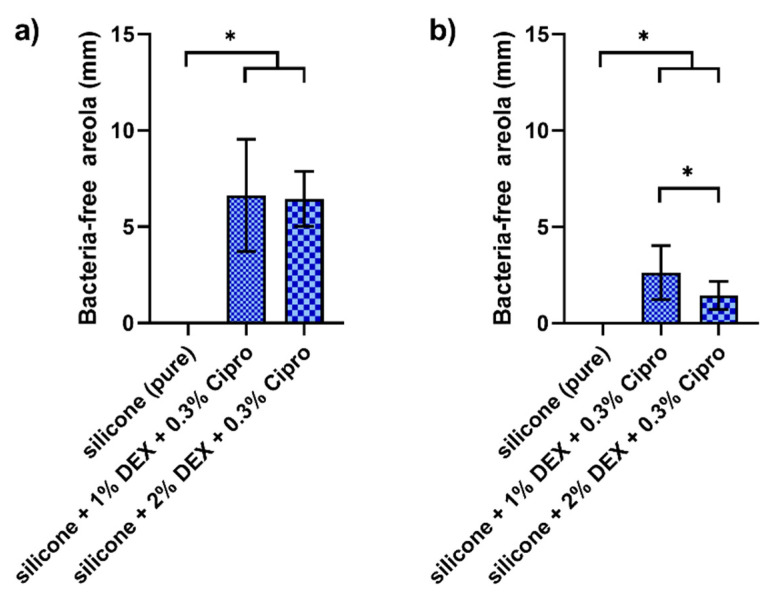
Diameters of bacteria-free areola of differently loaded silicone samples against *Staphylococcus aureus* (**a**) and *Pseudomonas aeruginosa* (**b**). Differences are indicated above the bars. For both bacterial strains statistically significant increases in bacteria-free areolas could be detected for both DEX and cipro loaded samples. For *S. aureus*, no difference in effectivity could be observed between 1% and 2% DEX, whereas for *P. aeruginosa* the bacteria-free areola was greater for 1% DEX. Data are given as box plots and significances are labeled with * *p* < 0.05.

To test for antibacterial activity of the DEX- and cipro-loaded UV–silicone, a standardized bacteria-free areola test was performed. For both bacterial strains, *S. aureus* (Figure 5a) and *P. aeruginosa* (Figure 5b), clear growth inhibition zones could be observed with silicone loaded with cipro and both 1% and 2% DEX. In contrast, no growth inhibition was detected for pure UV–silicone. The differences were statistically significant with *p* values from 0.006 t < 0.0001. Bacteria-free areolas were greatest for *S. aureus* with no difference between 1% and 2% DEX loading. For *P. aeruginosa*, inhibition zones were in general smaller than for *S. aureus*. Furthermore, the diameter of the bacteria-free areola was significantly smaller for 1% DEX than for 2% DEX (*p* = 0.03). Thus, silicone loaded with 1% DEX and 0.3% cipro exhibited the strongest antibacterial effect.

### 3.2. Drug Release Testing

The EECI model mass (n = 6) was 0.3288 g ± 0.0057 g on average and thus the implants contained a theoretical mean drug load of 3288 µg DEX (1%) and 986 µg cipro (0.3%).

**Figure 6:**
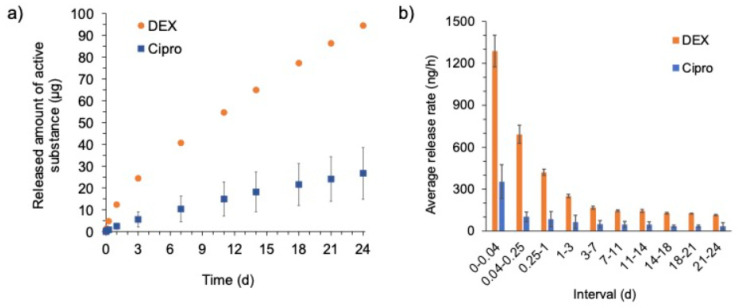
Mass of drug (DEX and cipro) released from the EECI model over 24 days (**a**) in total or (**b**) as average mass released per hour in the individual sampling intervals (n = 6, data are given as mean ± SD).

The release profiles of the active ingredients DEX and cipro from the EECI model are shown in Figure 6 in total (Figure 6a) and as an average mass released per time within the sampling interval (Figure 6b).

After one hour, averages of approximately 1.29 µg (standard deviation (SD) ± 0.11 µg) DEX and approximately 0.35 µg (SD ± 0.12 µg) cipro were released. Over the one-day period, means of approximately 12.33 µg (SD ± 0.41 µg) of DEX and approximately 2.39 µg (SD ± 1.26 µg) of cipro were released. On day 24 of the release assay, approximately 94.5 µg (SD ± 2.6 µg) DEX and approximately 26.7 µg (SD ± 11.9 µg) cipro had been released in total from the EECI model. The experiments were terminated at this time, even though drug release was not finished and had not reached a plateau. The release rate per day shows that, in general, slow release was faster initially for both DEX and cipro and then kept declining.

### 3.3. Microbial Contamination

An overview of the results of the test on microbiological contamination of the 3D printed EECI models is illustrated in Table 2. For the six tested samples incubated in soya-bean casein digest or thioglycollate medium, no growth of microorganisms was observed.

The observed turbidity or sediments in the positive control, as well as the clarity of the negative control, confirm the functionality of the test to detect aerobic bacteria. The bacterial growth of *Bacillus subtilis* remained well visible in the presence of the implant samples. Thus, the results of the test seem not to have been affected by the implant material itself.

### 3.4. Manufacturing of the Individualized External Ear Canal Implant 

**Figure 7:**
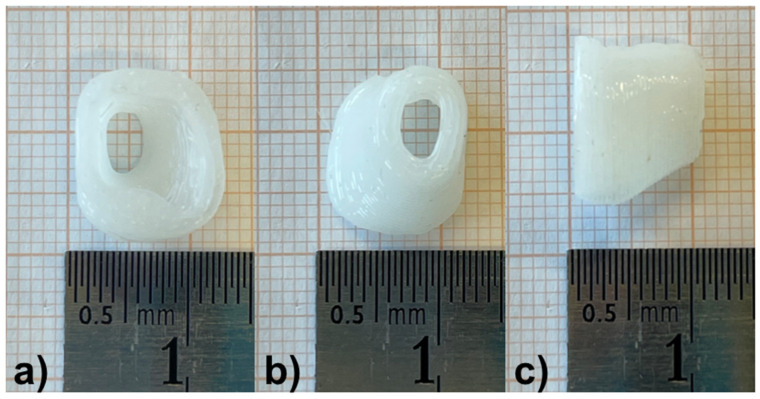
The patient-individualized DEX- (1%) and cipro- (0.3%) containing hollow EECI with a wall thickness of 1.5 mm after the printing process. (**a**) Top view of the side of the implant facing the opening of the EEC; (**b**) top view of the side of the implant facing the tympanic membrane; (**c**) side view of the implant showing its height.

One day prior to the surgery the patient-individualized EECI was printed as described above. Based on the results of the preclinical testing of the biocompatibility (MTT assay) and the bio-efficacy (TNFα-reduction test (DEX) and inhibition zone test (cipro)), the EECI was loaded with 1% DEX and 0.3% cipro. The patient-individualized EECI is shown in Figure 7. Based on the preoperative CBCT scan of the temporal bone, the EEC was segmented and the corresponding EECI was printed as described in detail above. After the printing process, the EECI was irradiated for 30 min in the UV crosslinker and transferred in an autoclaved pouch ready for transportation in the operating room.

### 3.5. Surgically Removal of the External Ear Canal Restenosis and Insertion of the External Ear Canal Implant

The use of the EECI was reported to the local ethics committee as an individual curative trial (Hannover Medical School). The patient and both her parents gave a written informed consent after in-depth consultation regarding the first-in-human use and the possible risks and potential complications.

**Figure 8:**
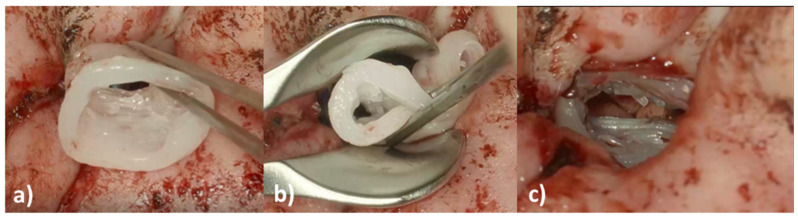
Intraoperative images of the insertion of the 3D-printed, individualized, drug-releasing EECI. (**a**) After surgically removing the stenosis of the EEC, the EECI was inserted in the right position according to the patient’s anatomy; (**b**) the texture of the implant was deformable for better handling while insertion with the forceps; (**c**) perfectly fitted implant in the EEC of the patient.

On the day of surgery, the patient underwent canaloplasty with removal of the restenosis on the right EEC under general anesthesia. Due to the patient history of three TM reconstructions a total reconstruction of the TM was performed using Tutoplast^®^ Fascia lata and perichondrium. Then the EECI was inserted using forceps (Figure 8a,b) with ease. The distal end of the implant was placed with some distance to the tympanic ring and the proximal end rested on the opening of the EEC (Figure 8c).

### 3.6. Clinical Case Follow Up

**Figure 9:**
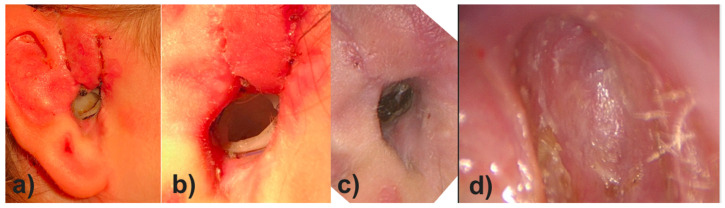
Postoperative images of the EEC of the patient during the follow up visits. (**a**) Averview of the auricle and EEC with the implant in situ two weeks postoperatively; (**b**) close-up of the EEC with the well adapted implant in situ three months postoperatively right before explantation; (**c**) the EEC four weeks after explantation of the implant (i.e., four months after implantation) without clinical signs of inflammation; (**d**) close-up of the EEC one year after surgery: the EEC shows a good patency and view on the TM after total reconstruction of the TM with Tutoplast^®^ Fascia lata and perichondrium.

The used medical-grade silicone is USP class VI but mainly aimed at research applications as well as direct skin contact for a maximum of 29 days, due to the lack of approval for chronic implantation. Since her family decided after 29 days to prolong the implantation duration, the EECI stayed in situ for three months. In the first six weeks postoperatively, the patient and her parents showed up in our outpatient clinic at two-week intervals and, after six weeks, for every month up to three months postoperative. Throughout the first three months postoperatively, there was no evidence of ear canal infections, otalgia, or otorrhea related to the EECI and the implant remained a good fit in the EEC (Figure 9a,b). The TM was visualized with the EECI in situ using an otoscope and there was no TM perforation, or any sign of middle ear infection during the follow-up period (no images shown; the endoscope diameter for photo documentation could not pass the EECI for visualization of the TM). Three months after surgery, the EECI was easily removed by cutting the implant at the posterior wall of the EEC and pulling it out with forceps (Figure 9c). The explanted EECI did not show tissue or epithelium attachment on its surface and the EEC was well epithelized and clear. In the following months, the patient was ordered into the outpatient clinic once ever quarter and reported no EEC infections or other otogenic symptoms. A follow up at one year postoperatively showed good patency of the EEC (Figure 9d) and the patient and her parents reported to be very satisfied with the treatment and outcome.

## 4. Discussion

Surgery for stenosis or atresia of the EEC remains one of the most challenging procedures in otology, mainly due to the relatively high postoperative complication rates [3]. Preventing restenosis after surgical repair is of utmost importance for postoperative success and patients’ satisfaction. The aim of this individual clinical treatment attempt was to help a patient with a long history of restenosis. For this, we introduced an alternative, improved method for EEC stenting after canaloplasty in patients with EEC stenosis. Our study is the first to use a 3D-printed, patient-individualized, drug-releasing EECI. In our first patient treated with an EECI, there was no evidence of ear canal infections or other otogenic complications related to the EECI throughout our follow-up period. Prior to our individual curative trial with the EECI, the patient suffered from persistent infections and restenosis. These complications did not recur during or after the EECI treatment.

There are several advantages of the use of our individualized, drug-loaded, hollow, mechanically flexible EECI in the prevention of restenosis after canaloplasty.

### 4.1. Silicone as Material to Additively Manufacture Implants

The basis of our novel treatment strategy is the 3D printing of drug-loaded silicone resulting in an implant perfectly fitting the patient’s needs. Silicone is an inert synthetic compound that comes in a variety of forms (oil, rubber, resin). Typically, heat-resistant and rubber-like, silicones are present in sealants, adhesives, lubricants, medical applications, cookware and insulation. Silicone is a polymer that contains silicon, combined with carbon, hydrogen, and oxygen and, in some cases, other elements. Accordingly, there is a range of chemically different silicones. Some silicone elastomers are used in biomedical applications due to their biocompatibility. In general, silicone has good UV resistance as well as excellent thermal and chemical resistance [21]. The fabrication of elastomers requires that the material cures or crosslinks through the process of molding. The silicone used here was crosslinked by UV light. The silicone material is USP class VI approved. The final formulation including the drugs was further examined regarding biocompatibility and implantability after printing and UV irradiation (see below).The individualization of the EECI via 3D printing allows the perfect fit to the patients’ anatomy. The implant design as a hollow body ensured that sound waves can be processed up to the TM and exudate from the wound can be rinsed out. The use of silicone to additively manufacture the EECI combines the beneficial characteristics of mechanical flexibility, allowing the insertion of the implant with ease, and resistance to the mechanical pressure of tissue. In order to use these properties of the silicone most appropriately for our application, the wall thickness of 1.5 mm was selected. This wall thickness was not only chosen due to its resistance to tissue pressure, but also because it enabled access to the tympanic membrane on the narrowest part of the EECI. A previous study reported that mechanical pressure modulates the remodeling phase of wound healing by altering the activity of matrix metalloproteinase-28 [14]. In addition, it has been proven that osteocytes act as mechanosensors and that mechanical pressure also leads to bone remodeling in the early stage [15]. Furthermore, besides exerting mechanical pressure through the EECI, avoiding postoperative inflammation is of utmost importance to prevent restenosis.

### 4.2. Dexamethasone 

Over the past decades, it is a widely held belief among clinicians that steroids significantly accelerate the resolution of symptoms by decreasing inflammatory conditions and eliminating or suppressing the granulation tissue formation. DEX is the most popular approach in the field of anti-inflammation [22]. However, although many studies highlight the therapeutic effect of DEX, factors such as the polarity of pro-drugs, the concentration to be applied or the delivery route affect its biological effects. In in vitro studies, the same DEX used as in this study was shown to be biocompatible up to 2000 µM (0.0784 mg/mL) [23]. In addition, this DEX was used for delivery through hydrophobic materials such as silicone in previous studies [24,25].

### 4.3. Ciprofoloxacin

Cipro is a fluoroquinolone with in vitro activity against a wide range of Gram-positive and Gram-negative microorganisms [26]. An FDA-approved topical otic suspension containing a combination of 0.3% cipro and 0.1% DEX remains the only marketed product approved for otic use [26]. Several clinical trials were conducted for cipro 0.3%/DEX 0.1% in the treatment of patients with acute otitis externa [27,28]. Among these, two studies found cipro combined with DEX were more effective than 0.3% cipro alone or 0.3% ofloxacin [29,30]. In the current study, we aimed not only to prevent acute infections postoperatively, but mainly the suppression of granulation tissue formation. Therefore, we decided to load our implants with a higher dose of DEX than cipro.

### 4.4. Biocompatibility of the EECI

Regarding the biocompatibility of the 3D-printed, drug-containing, and UV-irradiated (sterilized) samples, the test did not detect a cytotoxic effect of the UV–silicone itself or in combination with the drugs. However, the inclusion of 2% DEX and 0.3% cipro in the silicone slightly reduced the viability of the cells albeit not below the critical 70% cytotoxicity-limit. This may indicate that there is a limit for biocompatibility when further increasing the DEX concentration, which has to be considered before application in patients.

### 4.5. Bio-Efficacy of the EECI

The bio-efficacy testing in the TNFα-reduction test on DC cells detected a significant reduction in the cell stress reaction when 1% DEX, together with 0.3% cipro, were mixed in the silicone. The TNFα amount was reduced to a level not significantly differing from the unstressed NC and the included DEX control with a DEX concentration (60 µM, 23.5 µg/mL) frequently used to reduce inflammatory reactions. These results prove the biocompatibility and the efficacy of the DEX included in the silicone and clearly point to the anti-inflammatory effect of such an implant on the surrounding tissue. The TNFα amount in the supernatant of cells treated with eluates of silicone with 2% DEX was slightly higher than for the 1% DEX. In these wells, the number of cells was lower, which influenced the TNFα concentration/cell density. This is in accordance with the detected lower cell viability in the MTT assay for the 2% DEX silicone. That the higher amount of included DEX has not further reduced the TNFα release may indicate that a proportion of the included DEX was not bioavailable.

### 4.6. Microbial Contamination

Our 3D-printed samples were also tested for their antibacterial effect against the bacteria *Staphylococcus aureus* and *Pseudomonas aeruginosa* and their antibacterial effect could be verified. The concentration of 1% DEX and 0.3% cipro exhibited the strongest antibacterial effect on both strains. Based on these promising preclinical test results, the combination of UV silicone with 1% DEX and 0.3% cipro was chosen to treat the patient.

### 4.7. Drug Release

Regarding the drug release, both DEX and cipro showed a very slow release in the in vitro test over 24 days. Initially, a faster release rate was observed for DEX as well as for cipro. This kind of release behavior is called “burst release” or “fast initial release” and is sometimes desired in order to reach higher concentrations and thus therapeutic effects at an early stage of the therapy. However, it must be kept in mind, that in the dissolution test performed here the implant was in direct contact with medium (both on the luminal and abluminal side) for the entire time of the experiment. This differs from the clinical situation, where the implant has contact with the tissue only with its abluminal side, while the luminal side is in contact with air or fluid draining off the EEC. Therefore, reliable prediction of in vivo release is not possible from these in vitro data. It may be assumed that due to less fluid contact the in vivo release might be slower compared to the observed in vitro release and therefore achieving drug delivery over a very long period of time might be feasible using the developed implant.

For cipro, to date, there have been no silicone matrix-based implants for otological applications. Therefore, no comparison of the observed drug release of cipro with literature data for this special application is possible. Different working groups have prepared cipro or ciprofloxacin hydrochloride containing implants for the treatment of osteomyelitis or periodontitis based on different matrices like poly(DL)-lactide (PLA) [31], crosslinked high amylose starch [32], poly (epsilon-caprolactone) [33] or poly(lactide-co-glycolide) (PLGA) [34,35]. Recently, patient-specific, cipro-containing implants based on a matrix consisting of PLA, hydroxypropyl cellulose and microcrystalline cellulose for the treatment of bone defect diseases have been produced by Cui et al. [36] who report a limited burst effect for 3D-printed implants. The burst effect of the silicone matrix-based implants in our study was also relatively low and drug release was not finished after 24 days. However, due to the fundamentally different matrix used in this study, the comparability of the results of the in vitro release experiments is limited.

In contrast, usage of DEX as additive in silicone and an investigation of its release was described previously in ontological context, however not for EEC. Sircoglou et al. produced and investigated a DEX-containing silicon matrix (Kwik-Cast silicon) based Trans-Oval-Window implant [37]. The authors highlighted that silicone is a more suitable matrix for drug release at the oval window than resorbable biopolymers (such as PLGA or gelatine), as a higher drug load and a prolonged release can be achieved, which is favorable for chronic treatment. Additionally, several research groups have investigated the DEX release from silicone cochlear implants [25,38,39,40,41], all showing a burst followed by a prolonged release as also shown for our EECI. Liebau et al. observed silicone rods loaded with 0.1, 1, and 10% DEX after implantation into the cochlea of guinea pigs [31]. The silicon rods containing 1 and 10% DEX, respectively, lead to an initial burst release for up to 7 days and onwards to a steady-state phase that lasted until the end of the experiments (7 weeks). Therefore, our 1% DEX-containing EECI is in line with other devices used in preclinical ENT applications.

In future studies drug release from the implants should be studied in more detail and the testing period should be adapted to the expected in vivo implantation time. Furthermore, the impact of shape individualization on drug release will need to be addressed.

### 4.8. EECI Sterilization

The absence of microorganisms on inserted implants is essential to minimize the risk of infections resulting from the implantation into the EEC of patients. The ideal sterilization or disinfection of 3D-printed materials should effectively remove microorganisms, ensure that the sterilized material is non-toxic, while maintaining the physical and chemical properties of the material and biological activity of the included drug [42]. Ultraviolet (UV) irradiation is mainly used for the physical disinfection of material surfaces and transparent biodegradable scaffolds. It not only denatures nucleic acids and proteins directly with an effectiveness of 99.99% [43], but also transmits along a straight line and can be reflected or absorbed by the surface of an object. For the implants presented in this study, a method which works without fluid contact to avoid drug release during the sterilization process is necessary. Therefore, UV irradiation of the EECI was chosen which is an established method and the in vitro results did not indicate any contamination of the implants. Neither the silicone nor the drugs should be affected by the irradiation. But since it cannot be excluded that UV-irradiation does leads to changes in drug molecules the chemical structure of the released drugs after irradiation should be confirmed in future studies.

The advantages of the preoperative UV irradiation of the EECI were three-fold: (1) all the in vitro tests conducted in this study showed no changes in biocompatibility or bio-efficacy of the drug loaded silicone; (2) the drug release analysis demonstrated a drug release after UV irradiation of the EECI models; and (3) it is precisely this time-saving method that enables the manufacturing of an implant even in a preoperative workflow setting. For future clinical applications of our novel individualized printed implants the question of the sterilization technique will have to be considered in more detail.

The performed test on microbial contamination of our UV-irradiated EECI model did not detect any microbial contamination in all six tested EECI models. However, the test results are limited to the tested specimens and cannot easily be transferred to all printed products or the employed process in general, especially considering the relatively small number of EECI that were tested. A validated terminal sterilization or aseptic production process will have to be developed prior to further advancing an implant to the clinical routine. Although the tested EECI models only contained DEX, implants that are additionally loaded with the second drug (cipro) should provide similar results due to the antibacterial drug incorporated or potentially could even prevent growth of certain bacteria on the implant surface.

## 5. Conclusions

We believe that our 3D-printed, patient-individualized, drug-releasing EECI facilitates the prevention of restenosis after canaloplasty. This technique could not only be applied in patients with congenital EEC atresia or postoperative EEC stenosis, but also in patients with chronic external ear canal infections. This first individual treatment attempt cannot directly be applied to additional patients or pathologies. For further application of this novel approach of individualized drug releasing flexible implants, in-house manufacturing has to be employed. Risk and quality management procedures based on standardized operation procedures has to be established and approved by our ethics committee. The preparatory work is ongoing.

To verify the effectiveness of the EECI in comparison to classical treatment strategies, a controlled clinical trial is needed. Future developments should address the treatment duration as well, since it may be beneficial to keep the EECI in situ for more than 3 months as restenosis may occur up to 12 months postoperatively [44]. Today, the UV silicone is only approved for short-term use in the body (29 days or shorter). In the presented case, the patient and her parents decided to keep the EECI in situ for 3 months. Since there were no signs of any otogenic complications, prolonging the implantation duration after in-depth consultation with patients regarding the possible risks and potential complications can be considered.

## Figures and Tables

**Table 1:** Gradient conditions of the high-performance liquid chromatography (HPLC) method with eluent A (phosphate buffer containing triethylamine, pH 3.0) and eluent B (methanol), flow rate 0.4 mL/min.

Time (min)	Eluent A (%)	Eluent B (%)
0.00	80	20
3.00	80	20
12.00	35	65
12.10	80	20
16.00	80	20

**Table 2:** Results of the pre-test on microbiological contamination of the 3D-printed EECI after 14 days of incubation in culture medium. Bacterial growth was identified by the formation of turbidity or sediments.

Test Type	Culture Medium	
	Soya-Bean Casein Digest	Fluid Thioglycollate
negative control (pure media)	no microbiological growth	no microbiological growth
positive control (media + *Bacillus subtilis*)	microbiological growth	microbiological growth
test on antimicrobial effects (media + EECI + *Bacillus subtilis*)	microbiological growth	microbiological growth
test on microbiological contamination (media + EECI)	no microbiological growth	no microbiological growth

## Data Availability

All data are available upon request.
